# Prevalence, awareness and control of type 2 diabetes mellitus and risk factors in Chinese elderly population

**DOI:** 10.1186/s12889-022-13759-9

**Published:** 2022-07-19

**Authors:** Yaqiong Yan, Tingting Wu, Miao Zhang, Changfeng Li, Qing Liu, Fang Li

**Affiliations:** 1 Wuhan Centers for Disease Control & Prevention, 288# Machang Road, Wuhan, China; 2 Department of Epidemiology and Health Statistics, School of Public Health, Wuhan University, 115# Donghu Road, Wuhan, 430071 Hubei China

**Keywords:** Type 2 diabetes, Prevalence, Awareness, Control, Chinese elderly

## Abstract

**Background:**

Type 2 diabetes mellitus is an expanding global public health issue, especially in developing countries. This study aimed to investigate the prevalence, awareness and control rate of type 2 diabetes mellitus, and assess its risk factors in elderly Chinese individuals.

**Methods:**

The health screening data of 376,702 individuals aged ≥ 65 years in Wuhan, China, were collected to analyse the prevalence, awareness, and control rates of diabetes. Indices, including fasting plasma glucose and other biochemical indicators, were measured for all participants using standard methods at the central laboratory. Multilevel logistic regression analysis was performed to assess the key determinants of the prevalence, awareness, and control rates of diabetes.

**Results:**

The prevalence, awareness, and control rates of diabetes in the Chinese individuals aged ≥ 65 years were 18.80%, 77.14%, and 41.33%, respectively. There were statistically significant differences in the prevalence, awareness, and control rates by gender. Factors associated with diabetes prevalence were age, body mass index (BMI), and central obesity; while those associated with awareness and control were gender, education level, marital status, physical activity, alcohol consumption, BMI, and central obesity.

**Conclusions:**

Diabetes is an important public health problem in the elderly in China. The awareness and control rates have improved, but overall remained poor. Therefore, effective measures to raise awareness and control the rates of diabetes should be undertaken to circumvent the growing disease burden in elderly Chinese people.

**Supplementary Information:**

The online version contains supplementary material available at 10.1186/s12889-022-13759-9.

## Background

With a rapid economic development, dramatic changes in lifestyles, and an aging population, type 2 diabetes mellitus (T2DM) has become a leading public health problem globally, especially in developing countries [1–3]. According to the latest International Diabetes Federation (IDF), the global prevalence of T2DM in adults was 536.6 million people (10.5%) in 2021, and that there would be 783.2 million people (12.2%) living with diabetes worldwide by 2045 [4]. China, the largest developing country worldwide, contributes to one-fifth of the global population [5], and has shown increased prevalence of diabetes in recent years. A study by Yang et al. in 2007 identified that the prevalence rate of diabetes was 9.7% in 46,239 residents from 14 provinces in China [6]. The latest research data suggests 11% prevalence rate of diabetes in adults in China [7]. Taken together, the prevalence of diabetes is extremely high in China.

The number of individuals aged ≥ 65 years has approached 200 million in China. The results of several epidemiological surveys on prevalence of diabetes in the elderly population show that the individuals aged ≥ 65 years have a higher risk of diabetes than those in other age groups [1], and the incidence of diabetes in the elderly is increasing [8]. Therefore, it is crucial to assess epidemiological characteristics and risk factors of diabetes, and implement selective interventions and management for specific populations.

Studies suggest that severe diabetes can be alleviated with proper management and education [9–11]. The IDF recommends that the target for glycaemic control in patients with diabetes is fasting plasma glucose (FPG) < 7.0 mmol/L or glycosylated haemoglobin (HbA1c) < 7% [12]. However, a previous study showed that Chinese patients with diabetes have a poor blood glucose control, and only 26%–40% of patients with diabetes achieve the target [13]. A meta-analysis in mainland China indicated a mere 20.87% diabetes control rate [14]. Poor diabetes control rates impose a significant financial burden on individuals, families, healthcare systems, and countries. A study suggested that approximately $110 billion (12% of total health expenditure) was spent on the treatment and management of patients with diabetes in China in 2015 [15]. Thus, it is imperative to identify factors that affect blood glucose control to prevent and delay diabetes.

Several studies have investigated the prevalence, awareness, and control rates of diabetes in China [8, 16–18]. However, information on diabetes in large populations is scarce, and only few studies have assessed older adults facing a higher risk of developing diabetes. Therefore, this study aimed to assess the latest epidemiological characteristics of diabetes in Chinese individuals aged ≥ 65 years, including the prevalence and related risk factors, and to evaluate their understanding of diabetes and glycaemic control levels.

## Methods

### Study design and participants

This was a population-based, cross-sectional study in individuals aged ≥ 65 years in Wuhan, China. Wuhan had 1,278,902 elderly individuals in 2018 [19]. With a scheme launched by the Wuhan Municipal Government, the Wuhan Center for Disease Control and Prevention conducted health screening of 388,403 (30.37%) elderly individuals from 11 urban districts and 6 suburban districts between January 2018 and December 2018. All participants volunteered and signed informed consent forms. The inclusion criteria were that the participants were elderly individuals aged ≥ 65 years and who were permanent residents of Wuhan. After excluding 11,701 individuals with incomplete or missing data, 376,702 participants (96.99%) finally completed the entire study.

The study was reviewed and approved by the Ethics Committee of the Wuhan Center for Disease Control and Prevention (IRB#: WHCDCIRB-K-2018023). Written informed consent was obtained from all participants prior to the data collection.

### Data collection

The survey was conducted at local community health service centers in Wuhan, China. Each participant completed a health status questionnaire, and underwent body measurements and blood biochemical tests. Data were collected by trained medical staff at elementary healthcare centers.

A health status questionnaire was used to collect demographic information, including age (in years), gender (men, women), education level (elementary school and below, junior high school, technical secondary school or high school, and junior college or above), marital status (married, divorced, widowed, or single), history of diabetes (yes or no), physical activity (never, occasionally, sometimes, often, or always), smoking status (never, occasionally, often, or quit smoking), and drinking habit (never, occasionally, often, every day, or quit drinking).

Body measurements were obtained using standardised protocols. Participants’ height was measured in meters without shoes, and weight was measured in kilograms after removing heavy clothes. Body mass index (BMI) was calculated as weight in kilograms divided by height in meters squared (kg/m^2^). Waist circumference (WC) was measured by placing the measuring tape over the navel and horizontally circling the waist. Blood pressure (BP) was measured by a registered nurse in a seated position using a standard mercury sphygmomanometer or automatic manometer.

Blood samples were collected from all participants after an overnight fast of at least 10 h. All blood samples were analysed at the central laboratory, which successfully completed a standardisation and certification program. Fasting plasma glucose (FPG) levels were measured using the glucose oxidase procedure.

### Definitions

In this study, the diagnosis of T2DM was identified based on the Health Screening data. If one of the following three criterion was met, the subjects were regarded as T2DM: (1) FPG ≥ 7 mmol/L, (2) 2-h post-load plasma glucose ≥ 11.1 mmol/L, (3) self-reported previous diagnosis by healthcare professionals. The data were checked carefully by professionals to verify accuracy and completeness [20]. Awareness was defined as the proportion of individuals who reported a history of physician-diagnosed diabetes [15]. Control was defined as the proportion of patients with diabetes with FPG < 7.0 mmol/L, which indicated that self-reported history of diagnosed diabetes patients with FPG < 7.0 mmol/L at this physical examination were considered to have glucose control. Overweight was defined as BMI between 24.0 and 27.9, and obesity was defined as BMI ≥ 28.0. Central obesity was defined as a WC ≥ 85 cm for women and 90 cm for men according to the Healthy Adult Weight Determination in China (WS/T428–2013) [21].

### Statistical analysis

Data were double entered in Epi Data 3.2 after manual checking. Double entry was used to minimise data entry errors. Participant characteristics are described as mean ± standard deviation for continuous variables and number (percentage) for categorical variables. The associated risk factors for the prevalence, awareness, and control of T2DM were analysed using multivariate logistic regression. Adjusted odds ratios (ORs) and 95% confidence intervals (CIs) were calculated. A *P* < 0.05 was considered to be statistically significant. All statistical analyses were performed using SAS version 9.4.

## Results

### General characteristics of participants

The socio-demographic characteristics of individuals aged ≥ 65 years in Wuhan are shown in Table 1(Additional File 1). A total of 376,702 individuals were investigated in the study, of which 167,886 (44.6%) were men and 208,816 (55.4%) were women. The average age of participants in the T2DM group tended to be higher than that of participants in non-T2DM group. BMI, systolic blood pressure (SBP), WC, and FPG levels were higher in participants in the T2DM group than those in non-T2DM group. However, participants in the non-T2DM group had a higher diastolic BP (DBP) than those in T2DM group. The differences between T2DM group and non-T2DM group were statistically significant. A majority of the participants had low education levels. A majority of the participants were married, and never smoked or consumed alcohol.

### Prevalence, awareness, and control rates in different subgroups

The overall prevalence of T2DM in the Chinese individuals aged ≥ 65 years was 18.80% (Table 2, Additional File 2). There was a statistically significant difference in the prevalence of diabetes between men and women (χ^2^ = 208.414, *P* < 0.001). Of the individuals with T2DM, 77.14% were aware that they had diabetes, and 41.33% of them had their blood glucose levels under control. There were also statistically significant differences in terms of awareness and control rate by gender (χ^2^ = 379.454, *P* < 0.001; and χ^2^ = 95.683, *P* < 0.001, respectively). The results showed that individuals with a higher BMI and central obesity had a higher prevalence of T2DM than other individuals. The awareness and control rate for T2DM increased with age and education level, and was particularly high in individuals who exercised regularly. Moreover, individuals who quit smoking and drinking had the highest awareness and control rates, as opposed to those in other groups.

### Analyses of risk factors for the prevalence, awareness, and control of T2DM

In the multivariate analysis, older age, higher education level, physical activity, history of higher BMI, and central obesity were risk factors for the prevalence of T2DM, while smoking and drinking were negatively related to the prevalence rate (Table 3, Additional File 3). The education level, physical activity, quite smoking, quite drinking, history of central obesity, and history of high BMI were positively related to the awareness rate. In contrast, single marital status and current drinking were negatively related to the awareness rate. Further, older age, higher education level, physical activity, quite smoking and quite drinking were positively related to the control rate; while single marital status, current drinking, history of higher BMI, and central obesity were negatively related to the control rate. Additionally, women gender showed positive correlation with awareness and control rates.

## Discussion

This study investigated the prevalence, awareness, and control rates of diabetes in a large population in Wuhan, China. We found that 18.80% of the elderly residents had T2DM. In addition, 77.14% of the participants were aware that they had diabetes, and 41.33% of them had diabetes under control. These findings reveal that T2DM has been a major public health issue in China, and thus, suggests that more measures to control T2DM are needed.

The key observation from these new data was that the prevalence rates in individuals ≥ 65 in China was relatively higher than in some regional surveys in China reported in recent years (18.8% *vs*.16.9%) [22], and almost equaled the US population (20.4%) [23], despite the fact that overweight and obesity are far more prevalent in the United States. Basing on the fact that due to China's critical diabetes situation, T2DM is for certain to impose tremendous health care costs on elderly adults with diabetes [24], it is important to improve the awareness and control rates of diabetes in patients. In the present study, 77.14% of the participants were aware that they had diabetes, and 41.33% had controlled diabetes. According to previous studies in other regions in China, the awareness rate of diabetes was < 60%, and the control rate was approximately 30% [25, 26]. However, in some European nations and the US, which have relatively higher medical facilities, the national awareness and control rates of diabetes exceeded 70% and 50%, respectively [23, 27]. These data indicate that the awareness and control rates of diabetes in China have improved than those in the past, and diabetes health education programs have progressed. However, compared with rates in the developed countries, China would need more aggressive diabetes prevention and control.

The results of the present study showed that the prevalence of T2DM was higher in women than in men, which is consistent with results of other studies conducted in Asia [28–30]. Previous studies have suggested that the difference could be attributed to variability in risk factors for diabetes prevalence between men and women, or to women's longer life expectancy [31]. Women also had higher rates of diabetes awareness and control than men in the present study. This may be attributed to the fact that women use medical services more, have a higher awareness of social security, and have easier access to health services than men [32]. In addition, due to the higher prevalence of diabetes in women, those with diabetes may pay more attention to the disease, and thus, have higher awareness and control rates than men.

The present study revealed that older individuals with higher education levels had higher awareness and control rates of diabetes than those with lower education levels. This is consistent with the results of some studies [33–35]. Previous studies have confirmed that education level is positively correlated with the health literacy level in patients with diabetes [36]. The individuals with higher education are more self-conscious about health maintenance and know more about diabetes, and thus, consciously take steps to manage diabetes. A study based in the US has shown that obesity is associated with reduced awareness of diabetes, with obese individuals having lower blood sugar control levels than those with normal weight [37]. This finding is consistent with findings of the present study. Moreover, some observational and experimental studies suggest that unhealthy lifestyles, such as alcohol consumption, are associated with poor diabetes control [8], which is consistent with the results of the present study.

The present study found age, BMI, and central obesity to be risk factors for diabetes prevalence, which is consistent with results of previous studies on older individuals [21, 38]. With rapid development of the economy and urbanisation, the way of living has changed significantly in Chinese individuals. Chinese consumers have shown a sharp increase in consumption of meat and reduction in consumption of cereals. People are less physically active and adopt a sedentary lifestyle, which has increased the obesity rates in Chinese population and increased the risk of diabetes [39–41]. Previous studies have shown that smoking, drinking, and lack of physical exercise are closely associated with incidence of T2DM [42, 43]. However, these associations were not observed in the present study. This may be because we investigated participants' current behaviour rather than past behaviour, and some subjects may have changed their lifestyle after being diagnosed with T2DM, which may have affected the results.

To circumvent the high prevalence of diabetes and insufficiency of blood glucose control, the relevant government departments should continue to strengthen the prevention and management of patients with diabetes. First, to increase the early screening of patients with diabetes, early identification is crucial for the primary prevention of diabetes, especially in high-risk groups. Second, health workers should strengthen the management of patients with diabetes, regularly perform blood glucose testing, examine for complications, and impart self-management knowledge education in patients with diabetes. Finally, the government can adopt lifestyle intervention policies, lifestyle interventions can prevent or delay diabetes progress, advocating residents insist on physical exercise regularly, pay attention to weight, low salt, low fat, and low oil diet health education, which will help residents to develop a healthy lifestyle, improve lipid metabolism, maintain a healthy weight, and reduce the risk of T2DM.

This study has some limitations. First, due to the cross-sectional nature of the study, the causal relationship between T2DM and some risk factors could not be inferred; therefore, longitudinal studies are needed to verify the conclusions.Second, dietary habits, family history, and other chronic diseases associated with diabetes were not included as variables in the study.

## Conclusions

The study identified high prevalence of T2DM in Chinese elderly individuals, despite improvement in awareness and control, and highlighted the demographics compared to those in developed countries. In view of the large and growing elderly population in China, diabetes may cause great harm, and thus, it is necessary to strengthen the management of senile diabetes. The relevant departments should adopt appropriate policies and interventions to implement effective education programs. Additionally, individuals should adopt a healthy lifestyle, increase physical activity, and maintain healthy weight, as these measures would help in the management of diabetes and reduce the economic burden in China.

## Supplementary Information

**Additional file 1: Table 1.** Sociodemographic characteristics of adults aged 65 and older in Wuhan. Notes: T2DM, type 2 diabetes mellitus; SD, standard deviation; BMI, body mass index; SBP, systolic blood pressure; DBP, diastolic blood pressure; WC, waist circumference; FBG, fasting plasma glucose. * *P* < 0.05；** *P* < 0.01；***P < 0.001; Categorical variables were analyzed by chi-square test.

**Additional file 2: Table 2**. Prevalence, awareness, and control of T2DM among adults aged 65 and older in Wuhan. Notes: T2DM, type 2 diabetes mellitus; CI, confidence interval; BMI, body mass index. ^a^ Central obesity was defined as waist circumference 90 cm or more in men and 85 cm or more in women. * *P* < 0.05；** *P* < 0.01；***P < 0.001; Categorical variables were analyzed by chi-square test.

**Additional file 3: Table 3**. Risk factors for the prevalence, awareness, and control of T2DM. Notes: T2DM, type 2 diabetes mellitus; OR, odds ratio; CI, confidence interval; BMI, body mass index. ^a^ Multivariate logistic analysis. Model fittings were conducted using stepwise method with a threshold of 0.10 for variable inclusion in the model. ^b^ Central obesity was defined as waist circumference 90 cm or more in men and 85 cm or more in women. * *P* < 0.05；** *P* < 0.01；***P < 0.001; Variables that were not included in the final logistic regression model were marked with “-”.

## Data Availability

Data supporting the results of this study are not publicly available due to regulatory restrictions, but are available from the corresponding author on reasonable request.

## Abbreviations

T2DM: Type 2 diabetes mellitus
SD: Standard deviation
BMI: Body mass index
SBP: Systolic blood pressure
DBP: Diastolic blood pressure
WC: Waist circumference
FBG: Fasting plasma glucose
CI: Confidence interval
OR: Odds ratio
